# Exploring patient interpretation of an orthosis adherence checklist: A cognitive interview study

**DOI:** 10.1371/journal.pone.0344771

**Published:** 2026-03-18

**Authors:** Marjan Saeedi, Joy Christine MacDermid, Louis Ferreira, Sahar Johari, Mike Szekeres

**Affiliations:** 1 School of Physical Therapy, Department of Health and Rehabilitation Sciences, Faculty of Health Sciences, Western University, London, Ontario, Canada; 2 Clinical Research Lab, Roth McFarlane Hand and Upper Limb Centre, St. Joseph’s Health Centre, London, Ontario, Canada; 3 Department of Mechanical and Materials Engineering, Faculty of Engineering, Western University, London, Ontario, Canada; 4 School of Rehabilitation Sciences, Department of Occupational Therapy, Faculty of Health Sciences, Western University, London, Ontario, Canada; Iran University of Medical Sciences, IRAN, ISLAMIC REPUBLIC OF

## Abstract

Upper extremity orthoses are commonly used in rehabilitation to reduce pain, improve alignment, and support return to daily activities. These benefits depend on regular and correct use, yet adherence is influenced by personal, environmental, and device-related factors. Although a checklist of adherence-related items has been developed based on clinician expertise, it has never been evaluated by patients. Understanding how patients interpret these items is essential before the checklist can inform development of a patient-reported outcome measure (PROM) to support adherence. The aim of this study was to explore how people with lived experience of upper extremity pain and disability using upper extremity orthoses understand and respond to the items in an adherence checklist, and to identify areas requiring improvement. Cognitive interviews were conducted with adults who had used an upper extremity orthosis to evaluate a clinician-developed adherence checklist. Using a think-aloud approach and follow-up questions, participants described how they interpreted each item and selected responses. Directed content analysis was used to categorize interpretation issues related to clarity, relevance, and reference point. Thirteen participants were interviewed. Most items were interpreted as meaningful and easy to answer. However, several terms were perceived as confusing or overly technical, including “customizability,” “aesthetics,” and “undue strain.” Interpretation of some items depended on context; for example, “breathability” varied with weather, and “flexibility” depended on the intended function of the orthosis. “Affordability” was not relevant for many participants, indicating the need for a “Not applicable” response option. When asked about adherence, participants commonly described following instructions rather than a broader, multidimensional concept. Participants valued the intent and content of the items. Cognitive interviewing identified ways to improve wording, add contextual cues, and refine response options to better reflect diverse experiences. These refinements will support development of a patient-centred outcome measure for assessing adherence to upper extremity orthoses.

## Introduction

Upper extremity orthoses are widely prescribed in rehabilitation to reduce pain, improve joint alignment, and restore function in conditions ranging from musculoskeletal injuries to neurological impairments and post-surgical recovery [1,2]. These devices can be custom-fabricated, prefabricated, or custom-fitted, with design and selection influenced by clinical presentation, therapeutic goals, and available resources [3,4]. Since orthoses are a conservative treatment option, they are often preferred over surgical interventions for their potential to support recovery while avoiding procedural risks and higher costs [3]. However, these benefits depend on following prescribed wearing schedules and proper use, commonly referred to as adherence.

In rehabilitation research, “compliance” and “adherence” are sometimes used interchangeably but differ conceptually. Compliance refers to how well a patient follows a prescribed regimen, typically measured by hours worn compared to recommendations, and often implies a passive role. Adherence, by contrast, reflects a more active, patient-centred approach, including not only usage time but also engagement in follow-up, communication with providers, and integration of the device into daily life [5,6]. This broader construct respects patient autonomy and recognizes the influence of lived experiences. Because adherence is shaped by both external (e.g., device fit, comfort) and internal (e.g., beliefs, motivation) factors, understanding it requires exploring how patients make decisions about orthosis use. In the context of orthotic care, adherence refers to the extent to which a patient’s orthosis use aligns with agreed-upon recommendations from their healthcare provider, suggesting the World Health Organization’s broader definition for health behaviours [7]. Non-adherence to orthotic treatments has been reported at rates up to 70% [3,8], which can delay recovery, exacerbate symptoms, and increase healthcare utilization [7] and costs [9]. The WHO’s multidimensional model identifies social/economic, healthcare system, condition-related, therapy-related, and patient-related domains as key influences on adherence [7]. Although WHO’s multidimensional model was originally developed in the context of medication adherence, these domains reflect broader behavioural and contextual factors that are also relevant to rehabilitation interventions requiring sustained patient engagement, such as orthosis use. In other words, while this framework has informed medication adherence research, it remains underexplored for orthosis use, particularly in upper extremity care.

Despite the importance of adherence, current assessment methods, including clinician judgment [3,4], self-report logs [8,10], and device-embedded sensors [11–14], tend to capture primarily observable behaviours and often reflect more clinician-focused perspectives. These approaches may miss the personal, cognitive, and contextual factors that shape day-to-day adherence decisions. In orthotic care, adherence is closely linked to usability, defined here as the extent to which an orthosis can be used comfortably, effectively, and within the routines of daily life from the patient’s perspective, and involves shared decision-making between clinicians and patients throughout prescription, fabricating, and fitting. The lack of tools designed to assess usability- and usefulness-related factors that influence adherence may limit the therapeutic optimization of orthotic interventions [3].

Patient-reported outcome measures (PROMs) may focus on clinical outcomes which are the expected terminal impact of health interventions, or they may assess processes of care [15]. In this context, adherence is best understood as a patient-reported process or intermediate outcome that influences downstream clinical results rather than representing an endpoint itself [7,9]. The quintuple aims of quality improvement suggest that we should be thinking about patient experience and outcomes, the usability and impact on the clinicians, cost-effectiveness, and equity [16]. All of these dimensions are important in optimizing orthotic adherence and effectiveness. In this study, the quintuple aim is primarily reflected through its focus on patient experience and usability within orthotic care, while also informing clinician decision-making during measure development. At present, no PROM exists for fully assessing adherence to upper extremity orthoses in a way that recognizes the link between usability and adherence [3]. A measure of overall adherence in upper extremity rehabilitation includes clinician-rated items (e.g., whether assistive devices, orthoses/braces, or therapeutic adjuncts are used correctly) and patient-reported items (e.g., “Use my aids, orthotics/braces, home treatments”). However, this type of adherence measure neither isolates the orthosis as a specific item, nor identifies specific concerns that might be modified to enhance orthotic adherence. An ideal measure would therefore capture orthosis-specific experiences, usability-related factors, and patient perspectives that influence real-world adherence.

A structured checklist of factors that may influence adherence to upper extremity orthoses was recently developed by integrating evidence from a scoping review and the perspectives of clinicians and rehabilitation professionals who reviewed the evidence [17]. This checklist summarizes a wide range of specific factors that might affect whether patients use their orthoses as recommended. However, it was created entirely from research and professional viewpoints, and the patient perspective is lacking. Best-practice guidelines for PROM development, such as those outlined by the COSMIN initiative (COnsensus-based Standards for the selection of health status Measurement INstruments), emphasize establishing content validity through the perspectives of the target population to ensure items are relevant, comprehensive, and understandable to end users [15,18]. Accordingly, the objectives of this study were to: (1) examine how patients interpret potential items for a patient-based adherence measure, (2) use patients’ feedback to identify and improve areas requiring rewording or additional context, and (3) determine the overall relevance of the checklist approach and items to people with lived experience (PWLE).

## Materials and methods

### Study design and setting

This study used a descriptive qualitative design incorporating the principles of cognitive interviewing [19,20] to evaluate a checklist of factors influencing adherence to upper extremity orthoses and the clarity, relevance, and completeness of the items [19,21–23]. Cognitive interviewing is well established in the development of PROMs, enabling the examination of how individuals interpret, process, and respond to individual items, allowing researchers to identify unclear wording, conceptual gaps, redundancy, or problems with response options [19,20].

The study was conducted at the Roth | MacFarlane Hand and Upper Limb Centre (HULC) at St. Joseph’s Health Care London Hospital in London, Ontario, Canada. This hospital-based clinic provides prescription, fitting, and follow-up services for a wide range of upper extremity orthoses.

### Ethical considerations

This study was approved by the Western University Health Sciences Research Ethics Board (HSREB) (Project ID #126740). All participants provided written informed consent before participation and were informed that their involvement was voluntary and that they could withdraw at any time without consequence to their care.

### Participants and eligibility criteria

Participants were adults aged 18 years or older with a diagnosed upper extremity condition, musculoskeletal, neurological, or rheumatological, for which an orthosis had been prescribed. Eligible participants were either current orthosis users or had used one within the past six months and had at least two weeks of experience wearing the orthosis prior to the interview. The study included both custom-made and prefabricated devices, provided they were worn for therapeutic purposes. All participants needed to be able to provide informed consent and participate in an English-language interview. We excluded individuals with cognitive impairments that could interfere with informed consent or with recalling and discussing the items; those with severe communication difficulties that would impede meaningful participation; and those prescribed orthoses under mandatory post-operative or rehabilitation protocols in which orthosis use was required rather than based on patient choice (e.g., immediate post-surgical immobilization). These contexts were excluded because the study sought to explore the volitional aspects of adherence. Eligibility was assessed during recruitment and prior to the interview based on self-report, referral information when available, and the individual’s demonstrated capacity to provide informed consent and engage meaningfully in the interview.

### Sampling and recruitment

A purposive sampling strategy was used to achieve variation across multiple characteristics, including age, gender identity, diagnosis, type and duration of orthosis use, and socio-environmental context [24,25]. This approach supported the inclusion of a heterogeneous sample, enabling a richer exploration of how different experiences and backgrounds influenced participants’ interpretations of checklist items. Efforts were made to capture perspectives from individuals with diverse clinical conditions, orthotic types, and varying levels of prior experience with orthotic devices. Recruitment was monitored throughout the study to ensure representation across these characteristics.

Recruitment occurred between 01 June 2025 and 30 July 2025 and took place in person at St. Joseph’s Health Care London through the surgical and hand therapy clinics of HULC. Informed consent was obtained in written form prior to participation. Participation was entirely voluntary, and no compensation was provided. No identifiable personal information (e.g., full name, medical record number) was recorded during recruitment or linked to study data. Recruitment followed methodological guidance suggesting that approximately 10–15 participants are typically sufficient to identify most item-level comprehension issues in a single round of cognitive interviewing [19,26,27]. Data saturation was considered to be reached when three consecutive interviews yielded no new information [19,28,29]. In this study, saturation referred specifically to the absence of new concerns related to predefined categories of cognitive interviewing, including clarity/comprehension, relevance, reference point, response definition, perspective modifiers, and calibration across items [23], while remaining attentive to any additional issues raised by participants. To monitor this, interview transcripts were reviewed and analyzed immediately after each session to determine whether additional interviews were needed. Saturation was achieved within the final three interviews, at which point recruitment concluded.

### Data collection and management

Data were collected through one-on-one, semi-structured cognitive interviews with eligible participants, conducted by the first author (MS), who has formal training in qualitative health research and a professional background in orthotics and rehabilitation. Prior to each interview, participants received a Letter of Information and an Informed Consent form and were given as much time as they needed to review study details and ask any questions. Sessions were scheduled at the convenience of participants and held in a private consultation room to ensure comfort, confidentiality, and minimal interruption. Each interview lasted approximately 30–50 minutes. At the start of the session, participants completed a brief demographic form capturing age, gender identity, marital status, education, employment status, diagnosis, affected limb(s), type of orthosis, duration of orthosis use, prescribing/decision maker clinician, and perceived level of social support. These data were collected to describe the sample and to provide context for interpreting the findings. In addition to these items, participants rated their satisfaction with orthosis use and overall experience using five-point Likert scales. These ratings were used to support data triangulation by comparing participants’ ratings with their interview responses, helping to strengthen the credibility of the findings [30]. Following completion of the demographic form, participants were provided with a printed copy of the adherence checklist and instructed to read each item in turn.

The semi-structured cognitive interviews followed a guide (S1 Table) developed by the research team in line with best-practice recommendations for instrument development and cognitive interviewing methodology [20,31]. The guide was piloted with the first two participants to assess clarity and flow; as no major changes were required, their data were retained for analysis. The guide was iteratively reviewed throughout data collection to incorporate emerging insights, allowing flexibility to pursue meaningful content while maintaining consistency across interviews. Two core cognitive interviewing techniques were used during data collection: the think-aloud approach and verbal probing. During the think-aloud process, participants were encouraged to verbalize their thoughts as they read each checklist item and selected their response. This allowed the research team to observe participants’ immediate understanding, hesitation, confusion, or uncertainty as it occurred without introducing any cues from the interviewer. After each response, verbal probes were used to explore participants’ reasoning in more depth, including what specific terms meant to them, why they selected a particular response, and whether the item felt clear and relevant. Probing included both predetermined open-ended questions and additional spontaneous follow-up questions tailored to each participant’s response [31–33]. The interviewer used a combination of concurrent probing (while the participant was answering the item) and brief retrospective probing (immediately afterward) to explore comprehension, relevance, and how responses were mapped to personal experience. Using both approaches allowed us to capture both initial reactions and more thoughtful reflections, providing a fuller understanding of how participants interpreted each item [19,26,34]. At the end of each interview, participants were invited to share any general feedback about the checklist, including whether they felt any important factors were missing or whether any items felt repetitive, unclear, or difficult to answer. Field notes were recorded immediately after each interview to capture contextual observations, such as hesitations, tone of voice, body language, or moments of emphasis, that could enrich interpretation. The interviewer also documented misinterpretation, requests for clarification, or difficulty mapping experiences to the provided response options [22]. All interviews were audio-recorded with participant consent and transcribed verbatim by the first author (MS) and checked for accuracy by another author (SJ) through comparison with the original recordings. To enhance analytic sensitivity and accuracy, transcripts were verified through multiple rounds of active listening. All transcripts were de-identified by removing names and any other potentially identifying details before analysis. Transcripts and field notes were stored as password-protected electronic files on secure institutional servers accessible only to the research team.

### Data analysis

All interviews were transcribed and organized in a question-by-question format corresponding to each adherence checklist item. A directed (deductive) qualitative content analysis approach [35] was used to identify and categorize issues affecting how participants interpreted and responded to each item. The analysis was deductive, guided by a previously established coding system developed by one of the study authors (JM) [23]. This system classifies interpretation issues into six categories: 1) Comprehension/clarity, 2) Perspective modifiers, 3) Reference point, 4) Calibration across items, 5) Inadequate response definition, and 6) Relevance [23].

The first author (MS) and another author (SJ) independently coded all transcripts, assigning one or more of these categories to each checklist item where applicable. Notes were also made on specific terms or concepts that were unclear, redundant, missing, or otherwise problematic. Following independent coding, the coders compared results, discussed discrepancies, and reached consensus on final coding decisions. Findings were summarized at the item level, indicating the type(s) of interpretation issues, frequency of occurrence, and illustrative participant quotations. Patterns were then examined across items to identify broader conceptual or structural concerns and potential gaps in content. Data collection and analysis proceeded concurrently, enabling the research team to monitor for emerging issues.

### Researcher positionality

In keeping with the principles of cognitive interviewing, we approached this study with an emphasis on careful examination of how participants understood and interpreted each checklist item through respectful and open dialogue [19,20]. The first author (MS), a certified orthotist-prosthetist and PhD candidate with international clinical and qualitative research experience, maintained reflexive journaling to account for professional assumptions. Another author (SJ) a PhD and occupational therapist, contributed expertise on patient–therapist interactions and functional outcomes. The senior author (JM), a physical therapist and hand therapist, and qualitative researcher with over 17 years of clinical and research experience, provided methodological and clinical guidance. Ongoing reflexivity and team discussion were used to help minimize bias in item-level interpretation and to ensure that decisions about pre-defined themes and options were based on participant input rather than researcher assumptions.

### Rigour and trustworthiness

To enhance methodological rigour, we used analyst triangulation, with two team members independently reviewing and coding all interview transcripts using the predefined cognitive interviewing framework. Coding results were compared, and any differences were resolved through discussion until agreement was reached. Reflexive memos were used throughout the study to record methodological decisions and to monitor how the researchers’ professional backgrounds and assumptions might influence judgments about item interpretation. Regular team meetings were held to review patterns across items, confirm final item actions, and ensure that all revisions were grounded in participant feedback rather than researcher assumptions. Data triangulation was also used by comparing participants’ interview responses with their ratings of satisfaction with orthosis use and overall experience. This helped confirm the consistency of participants’ views across different data sources. Together, these strategies supported the consistency, transparency, and credibility of the item evaluation process [36].

## Results

### Participant characteristics

Table 1 presents the demographic information of the participants. The sample consisted of 13 participants, primarily of adults over 50 years of age (61.5%) and was predominantly female (61.5%). Just over half of the participants were married (53.8%), and the majority used a static orthosis (76.9%). Overall satisfaction with orthosis use was high, with most participants reporting being very satisfied or satisfied (92.3%), and overall experience with orthoses use was rated as good or very good by most participants (92.3%). The final three interviews that confirmed saturation included participants with differing upper extremity diagnoses, orthosis types, and demographic characteristics, supporting that no new concerns emerged across varied participant profiles.

**Table 1:** Participant demographic characteristics (n = 13).

Characteristic	n	%
**Sex**		
Female	8	61.5
Male	5	38.5
**Age (Range: 26–78 years old)**		
Above 50 years	8	61.5
Below 50 years	5	38.5
**Marital status**		
Single	3	23.1
Married	7	53.8
Divorced	2	15.4
Widowed	1	7.7
**Education**		
Secondary school	3	23.1
College diploma	8	61.5
University degree	2	15.4
**Employment status**		
Employed	6	46.1
Unemployed	2	15.4
Retired	5	38.5
**Hand dominance**		
Right	12	92.3
Ambidextrous	1	7.7
**Orthosis type**		
Static	10	76.9
Dynamic	1	7.7
Functional	1	7.7
Combined device	1	7.7
**Satisfaction with orthosis use**		
Very satisfied	6	46.1
Satisfied	6	46.1
Neutral	1	7.7
**Overall experience**		
Very good	5	38.5
Good	7	53.8
Neutral	1	7.7

### Overview of findings

Across the 11 domains of the adherence checklist (Comfort, Fit, Durability, Customizability, Breathability, Flexibility, Weight, Aesthetics, Cleaning/Maintenance, Affordability, and Overall Satisfaction), most items were interpreted as intended and judged relevant to participants’ lived experiences with upper extremity orthoses. Several domains -Comfort, Durability, Cleaning/Maintenance, and Overall Satisfaction- were consistently understood without difficulty, requiring no changes. The most frequent issues were concentrated in a smaller set of items and mapped onto the theme frameworks, particularly Comprehension/clarity, Relevance, and Reference point. These issues reflected technical or formal wording, context-dependent interpretations, and circumstances in which the item was not applicable. Some problems were identified under Inadequate response definition, or Perspective modifiers, and when present, they did not compromise item utility. The application of these themes enabled systematic classification of these issues and guided targeted refinements to improve item clarity, contextual fit, and response option inclusivity.

In addition to verbal responses, field notes captured initial reactions when the printed checklist was first presented. Field notes documented that 11 of the 13 participants paused, re-read, or commented when first presented with the checklist, describing it as “a lot to go through” or appearing lengthy. While all participants completed the task, these reactions suggest that future formatting and presentation may influence initial acceptability.

### Checklist item performance overview

All 11 checklist domains were reviewed using think-aloud and targeted probes. Most items were interpreted as intended and considered applicable to participants’ experiences. Where issues arose, they were classified according to five themes. These themes included comprehension/clarity, relevance, reference point, inadequate response definition, and perspective modifiers. Each of these themes is summarized below.

1. **Comprehension/clarity**

Some items contained terminology that participants found technical, formal, or unfamiliar.

“Customizability” was misinterpreted by several participants as colour or style rather than functional tailoring. As one participant asked: *“That… customizability… I don’t know if I… like a green splint? … how do you customize it?” (P10)*. Another participant stumbled repeatedly: *“Customability. Customizability. Customizability.” (P16).*

“Weight” was generally understood, but the phrase “undue strain” was unfamiliar. *“What’s undue? … ‘Undue strain’ is a bit weird.” (P11)*. Another suggested: *“Apparently not a good wording of a question. That needs to be reworded a little bit.” (P16)*.

“Aesthetics” was not immediately accessible; most participants hesitated before answering, and one explicitly requested clarification of its meaning. *“… overall aesthetic? The E was kind of like an O or a C on me. I could not read the word at first.” (P07)*. Another said: *“The only one is one word that … I don’t know what it means, but it’s just me… Aesthetic…” (P08).*

These patterns suggest that simplifying language and offering concrete examples would improve clarity.

2. **Relevance**

Some checklist items were not relevant to all participants’ circumstances.

“Affordability” was commonly judged irrelevant for those with insurance coverage: *“I didn’t pay for it… It was covered by the insurance.” (P06)*. Another explained: *“Very good, because it’s covered under OHIP.” (P11)*. Similarly, one participant noted: *“It didn’t cost, there was no cost involved to me… it’s not relevant actually to my situation.” (P17)*.

Although the concept was understood, participants highlighted the need for a “Not applicable” option.

3. **Reference point**

Some items were interpreted differently depending on environmental or treatment context.

“Breathability” responses often depended on climate or activity. *“These get so smelly right now. I have to get a new one every couple of days.” (P16)*. Another reflected: *“It’s not. It’s very sweaty and stinky and makes sleep hard for the heat and the immobility.” (P09)*. A third added: *“When I go for walks and I put it on, on very hot days, it’s just like a steam sauna on your arm.” (P19).*

“Flexibility” was interpreted relative to expectations of movement. Some saw restricted motion as a flaw: “*With the splint, you can’t move your finger like you should.” (P06)*. Others acknowledged that immobility was the splint’s purpose: *“It’s intended not for my hand to move. If it does move, then it’s not doing its job.” (P17).*

These examples highlight the importance of clarifying intended purpose and context in item wording.

4. **Inadequate response definition**

No major mismatches were found between participants’ intended answers and available response options, aside from “affordability”, which is mentioned in relevance section.

5. **Perspective modifiers**

In some cases, responses were shaped by broader personal or relational factors. For example, satisfaction ratings were occasionally influenced by trust in clinicians rather than the orthosis itself: *“I’m not the expert, so I trust, you know, them to know what they’re doing.” (P13)*. While not problematic for item clarity, these modifiers illustrate how lived context may influence scoring.

### Summary of recommended actions

Of the 11 checklist domains assessed:

- 4 are recommended for retention without change (Comfort, Durability, Cleaning/Maintenance, Overall Satisfaction)
- 4 are recommended for rewording (Fit, Customizability, Weight, Aesthetics (plain-language alternatives may improve accessibility)).
- 1 is recommended for retention with a contextual cue (Flexibility).
- 1 is recommended for retention with an environmental example (Breathability).
- 1 is recommended for retention with a “Not applicable” response option (Affordability). (Table 2)

**Table 2:** Checklist items, themes, recommended action, and representative participants’ quotations.

Checklist item	Theme	Proposed action	Representative participants’ quotations
Comfort	–	Retain	Interpreted as intended; variation reflected true experience.
Fit	C	Reword to clarify snugness vs. coverage	“Adjustable things that I could make tighter…” (P10)
Durability	–	Retain	Clear; examples (Velcro/plastic) given
Customizability	C	Reword (simpler term + example)	“Like a green splint? … how do you customize it?” (P10); “Customability. Customizability.” (P16)
Breathability	RP	Retain with environmental cue	“Pretty sweaty and stinky…” (P09); “Steam sauna on your arm.” (P19)
Flexibility	RP	Retain with context note	“Can’t move your finger like you should.” (P06); “It’s intended not for my hand to move.” (P17)
Weight	C	Reword (remove “undue strain”)	“What’s undue? … ‘Undue strain’ is a bit weird.” (P11); “Needs to be reworded.” (P16)
Aesthetics	C	Retain (but plain word may help)	Hesitations/clarifications; “The only one is one word that … I don’t know what it means… Aesthetic…” (P08)
Cleaning/Maintenance	–	Retain	Clear and specific
Affordability	R, IR	Retain with “Not applicable”	“It was covered by the insurance.” (P06); “It didn’t cost… not relevant.” (P17)
Overall satisfaction	PM	Retain	Understood; sometimes influenced by provider trust

C = Comprehension/clarity; R = Relevance; RP = Reference point; IR = Inadequate response definition; PM = Perspective modifiers.

### Participant definitions of adherence

At the end of each interview, participants were asked how they personally defined “adherence” and what “using it as prescribed” meant to them. Responses showed both alignment with clinical expectations and important nuances in interpretation. Several participants defined adherence as following the therapist’s or surgeon’s instructions precisely: *“That I should be using it as been told by my therapist. And, yeah, to get better, I have to adhere to whatever was guided to me.” (P12)*. Others emphasized the need for flexibility and individual adaptation, highlighting that strict time-based prescriptions may not be realistic for all patients: *“You can’t tell somebody to wear it a certain time, because everybody’s different… I could not wear it a very long time [because of swelling], but maybe after a couple weeks, I could wear it longer.” (P15)*. For some, adherence was framed around personal responsibility and future consequences, extending beyond the splint itself: *“Any kind of medical device… knowing what the consequences could be if I don’t use it. … If I don’t adhere to following the proper procedures, that is going to affect my ability to function down the road.” (P17)*

Together, these responses illustrate that while most participants associated adherence with following prescribed guidelines, many also integrated personal context, physical limitations, and long-term motivation into their definitions. This variation underscores the importance of using patient-centred wording in adherence measures and ensuring that items capture both prescriptive and adaptive dimensions of splint use.

## Discussion

This study determined that items generated from a usability-based adherence checklist, developed through the integration of evidence and clinical expertise, was seen as comprehensive and relevant to PWLE, but required greater attention to clarity and health literacy issues for a PROM version. Through cognitive interviewing, we identified areas of clarity as well as potential sources of misinterpretation across the 11 domains of the checklist. Overall, participants confirmed the importance of most items but highlighted the need for refinements to improve clarity, contextual fit, and inclusivity.

### Patient interpretations of technical language

Although most items were understood as intended, several participants struggled with technical wording. For example, terms such as “customizability” or “undue strain” were described as overly formal or confusing. Similarly, the item “aesthetics” often required clarification, with many participants pausing, rereading, or asking for its meaning before responding, and “hygiene” was interpreted by one participant as referring to personal cleanliness rather than device maintenance. These findings suggest that while the underlying constructs were meaningful, wording in technical or professional terminology created a barrier, therefore addressing this misinterpretation is important to ensure accessibility across varying education levels. In this study, wording was considered for refinement when participants demonstrated hesitation, requested clarification, or interpreted an item differently than intended during cognitive interviewing. Prior research on PROM development has emphasized the importance of plain language and patient-centred wording, as patients may interpret terms differently from clinicians [15,37,38]. This aligns with work by Azad et al. [39], who demonstrated that difficulties in interpreting PROM items can be linked to health literacy, using education level as a proxy in their study. Their findings showed that when participants struggle to interpret an item’s intent, they may rely on guessing or personal assumptions, ultimately compromising the accuracy of their responses. Such misinterpretation can distort the measurement properties of a PROM, highlighting the need to ensure that terminology is appropriate for individuals across diverse literacy levels. Similar concerns were highlighted in the studies by Katz et al. [40] and Gruson et al. [41], and Trotter at al. [42], which showed that health literacy and the ability to understand PROM wording directly influence how patients score items, meaning that misinterpretation can alter the PROM results themselves. Our findings reinforce the need to replace technical phrasing with everyday language and, where necessary, provide examples to anchor meaning.

### Contextual influences on item interpretation

Several items were interpreted relative to situational or environmental context. “Breathability” was judged against seasonal conditions, with participants describing excessive sweating during hot weather, while showing no problem during cold seasons. “Flexibility” was also context-dependent: some participants saw limited motion as a problem, while others understood immobility as the intended purpose of their orthosis. One participant noted that “flexibility” might be better framed in terms of whether the orthosis allowed them to complete daily routines such as cooking, dressing, or caring for children. This perspective highlights that adherence is less about absolute mobility and more about whether the orthosis supports meaningful participation in every day’s roles. Moreover, these findings showed that patient interpretations often draw on immediate lived experience rather than the intended construct definition, underscoring the importance of contextual cues in item wording. These findings are consistent with research showing that people often answer PROM items based on their personal experiences and the situations they bring to mind. Work by Rapkin and Schwartz [43] demonstrates that individuals draw on their own frame of reference when interpreting items, meaning their responses reflect lived experience rather than the abstract construct the PROM is meant to measure. Vanier et al. [44] similarly argue that the meaning of terms used in surveys or questionnaires must be clearly defined, because different interpretations can shift how respondents understand an item and, in turn, how they score it. Another study [45] has also shown that the surrounding environment shapes how people respond to PROM items. For example, in this research with young people they found that the population rated their mobility according to the specific environments they moved through. A similar pattern was evident in our study: younger adults tended to interpret “flexibility” in relation to active roles such as exercising or completing busy daily routines, while older adults tended to associate the term with safety and stability rather than movement. These age-related differences illustrate how contextual expectations influence item interpretation. Taken together, these findings support the argument made by Hawkins and colleagues [46] that the validity of any instrument depends on whether it produces data that are appropriate, meaningful, and useful for its intended purpose. Our results, alongside previous evidence, show that patients rely on their immediate lived experience when interpreting PROM items. These findings suggest that context-dependent interpretation is an expected feature of patient-reported data, highlighting the need for a PROM that accommodates diverse experiences through clearer wording and examples to capture relevant responses. This underscores the importance of providing clearer contextual cues and examples within item wording to ensure that the intended meaning is shared across different ages, roles, and environments.

### Adherence as a dynamic and evolving process

Interpretations of items referring to “extended periods of time” also revealed important temporal considerations. Some participants described initially wearing their orthosis inconsistently, with adherence changing over time as swelling subsided, comfort increased, or they learned compensatory strategies. This suggests that perceptions of comfort, fit, and wearability may change over time and that adherence is not static but evolves with adjustment and experience. Measures of adherence may therefore need to specify whether responses should reflect early use or longer-term wear, or alternatively, capture both perspectives.

A study by O’Brien [5] examining strategies to improve adherence in hand therapy also highlighted “therapy-related interventions,” including helping patients adapt their activities without compromising orthosis use, as an important facilitator of sustained adherence. This aligns with our finding that participants described shifts in adherence once they learned compensatory movements or strategies that allowed them to carry out daily tasks while wearing their orthosis. Moreover, evidence from a systematic review of upper extremity interventions also supports the idea that adherence can change with experience, particularly when patients receive behavioural support such as self-efficacy-based education or clear communication from clinicians. Although the review does not suggest that adherence naturally improves over time, it highlights that adherence is responsive to behavioural and contextual factors [47]. These findings highlight the importance of ensuring that time-related items in our PROM are worded clearly and specify the period of reference that respondents should consider. Our cognitive interview findings showed that some participants drew on their immediate lived experience, such as early discomfort, later adjustment, or changes in swelling, when interpreting these items. Clarifying whether an item refers to early wear, current wear, or typical wear over time will help reduce inconsistent interpretations and improve the accuracy of the adherence scores captured by the PROM.

### Relevance and applicability of items

Certain items were not universally relevant, particularly “affordability”. For participants with full insurance coverage, this item was considered inapplicable. Although all participants in this study had insurance coverage, some anticipated that affordability could be a major barrier for individuals in other contexts or healthcare systems. This variability suggests that some items require a “Not applicable” response option to avoid forcing participants into ratings that do not reflect their experience and to ensure the measure is sensitive to health system differences. Similar issues have been reported in PROM development, where socioeconomic or health system differences shape item relevance [48]. Retaining “affordability” as a domain is important for inclusivity, but flexibility in response options is essential. Research supports this interpretation. For example, a study by Bonsel et al. [49] showed that socioeconomic factors can influence PROM scores and that these factors remain significantly associated with PROM outcomes even after statistical adjustment. This reinforces that items linked to socioeconomic conditions, such as affordability, may not be interpreted uniformly across patients and therefore require inclusive response options to avoid measurement bias. Similarly, Al Sayah and colleagues [50] have emphasized that PROMs must be selected and interpreted in relation to the health system and cultural context in which they are used. This is directly relevant to our setting, because all participants in our Canadian sample had insurance coverage for their orthosis, they naturally interpreted affordability differently than patients in settings where orthotic costs are not covered. This highlights the importance of designing items that are flexible enough to be meaningful across diverse health systems and socioeconomic conditions, while still capturing the construct of interest.

### Patient reflections on adherence

At the conclusion of the interviews, participants were asked what adherence meant to them. When asked to define adherence, almost all participants required clarification, with only one participant (who had a healthcare background) readily offering a definition. Once rephrased as “using it as prescribed,” participants understood and responded. Their responses emphasized practical and personal interpretations rather than abstract definitions. However, interpretations often reflected compliance with professional instruction rather than the broader, multidimensional concept of adherence described in the literature [7]. These findings suggest that patients perceive adherence as context-specific, negotiated around daily routines, symptoms, and personal circumstances, rather than a fixed obligation. This reflects the broader conceptualization of adherence described earlier in this study, where adherence extends beyond simply following instructions to include personal meaning, daily integration, and shared decision-making. Importantly, many did not recognize that factors such as aesthetics, comfort, or social support could be legitimate aspects of adherence, and some reported never considering adjustments or requests (e.g., different colors, design changes) that might improve their use or adherence. This gap highlights the educational potential of PROMs, by framing adherence as a multidimensional ongoing shared decision-making process. This tool could empower patients to discuss and negotiate factors affecting their use, rather than perceiving orthosis wear as a rigid directive.

### Implications for future refinement

The findings suggest practical directions for refining the adherence checklist. Technical terms may benefit from being reworded in plain language, contextual cues could be added where interpretation varied by situation or device type, and response options may need to accommodate variability in health system coverage. Beyond item wording, these interviews revealed that adherence itself was understood by PWLE in narrow terms, largely equated with compliance. Clarifying the broader construct of adherence, and embedding this in patient-friendly definitions, may support both measurement accuracy and patient engagement. This aligns with Gardner’s [9] assertion that definitions of concepts must be derived and analyzed within the context in which they are used [51], suggesting that adherence may not carry the same meaning across different patient groups or clinical environments. Incorporating this perspective into PROM refinement helps ensure that item definitions reflect real patient understanding rather than hypothetical or researcher-driven interpretations. Such an approach acknowledges that adherence is not only about duration of wear but also about whether orthoses support meaningful daily functioning and role performance.

### Strengths and limitations

A key strength of this study is its use of cognitive interviewing with patients who had direct lived experience of using upper extremity orthoses (PWLE). This approach ensured that the evaluation of the checklist items was grounded in patient perspectives rather than relying solely on clinician assumptions. The think-aloud method, combined with targeted verbal probes, provided detailed insight into how participants understood, interpreted, and responded to each item. Another strength is that the sample included variation in age, diagnoses, and orthosis types, which allowed us to identify a range of interpretation challenges and context-dependent issues.

Several limitations should also be noted. Although the sample included some diversity, it was relatively small and drawn from two clinical settings, which may affect the transferability of the findings to other populations and contexts. As no compensation was provided, participation was voluntary and may reflect the perspectives of individuals who chose to take part. All participants had access to insurance coverage, which limited the ability to fully explore how affordability influences adherence in less well-supported health systems. Because all participants were recruited from a single geographic region, cultural and health system differences in other settings may not be fully captured. Although a purposive sampling strategy was used to achieve variation, the sample may not fully represent the breadth of experiences of individuals from underrepresented populations. In addition, while a six-month timeframe for prior orthosis use was chosen to reduce recall bias, some degree of recall inaccuracy may still be possible among former users. The six-month timeframe was chosen to ensure that participants had sufficient experience with orthosis use to reflect meaningfully on the items.

### Future directions

Future work should build on these findings through additional stages of the PROM development process. While the interviews provided valuable insights into item clarity and relevance, they did not directly establish whether the proposed revisions will improve psychometric performance; this requires further testing in later phases. Larger and more diverse samples should be included to enhance transferability and capture variation in patient experiences across socioeconomic and cultural contexts. Cross-cultural adaptation may also be needed to ensure wording and concepts remain meaningful across different health systems. Finally, subsequent steps in item refinement and psychometric evaluation will be critical to confirm the reliability, validity, and responsiveness of the revised adherence measure.

### Implications for research and practice

From a measurement perspective, these findings strengthen the checklist’s content validity by ensuring that each item is both understandable and meaningful to the intended respondents. This is critical for any tool intended to inform clinical decision-making, as unclear or irrelevant items can compromise data quality. Clinically, the study underscores the importance of incorporating patient feedback early in tool development, particularly for devices that may carry unique functional, sensory, or contextual considerations. The intent of this work was to develop a comprehensive PROM to assess adherence to upper extremity orthoses. This approach was chosen to maximize applicability across different joints, orthosis types, and diagnoses recognizing that while experiences of use may vary, the broader concept of adherence may share common elements across contexts. Future work may explore whether additional refinement or validation is needed for specific joints, orthosis designs, or diagnoses.

## Conclusion

In conclusion, this study provides important insights into patients’ experiences with an adherence checklist for upper extremity orthoses. By highlighting both the strengths of the existing items and the areas requiring refinement, the findings contribute to the development of measures that are clear, relevant, and inclusive of patient perspectives. Continued research and testing are essential to ensure that future tools for assessing upper extremity orthoses adherence are both evidence-informed and practically meaningful in rehabilitation care.

## Supporting information

S1 Table Cognitive interview guide used to evaluate patient interpretation of checklist items. (DOCX)
