# Supplementary material for: Exploring patient interpretation of an orthosis adherence checklist: A cognitive interview study
Source: PLoS One. 2026 Mar 18;21(3):e0344771. doi: 10.1371/journal.pone.0344771 (PMC12998876; doi:10.1371/journal.pone.0344771)
Supplement: S1 Table — (DOCX) [file pone.0344771.s001.docx]

S1 Table: Cognitive interview guide used to evaluate patient interpretation of checklist items.

| **Interview Question** |
| --- |
| **Introduction to the Checklist (Think-Aloud Protocol)** |
| 1. **I am going to give you an adherence checklist that healthcare providers developed based on their observations. As you go through the checklist, I’d like you to read each item aloud and tell me what comes to mind.** |
| 1. Does this statement make sense to you? |
| 1. How do you interpret this statement in relation to your experience with the splint (orthosis)? |
| **Evaluating Specific Factors on the Checklist** |
| 1. **Let’s go through key sections of the checklist. Please share your thoughts as you read each item.** |
| **Comfort & Fit** |
| - 1. How do you decide whether your splint (orthosis) is comfortable enough to wear? |
| - 1. Does this checklist item reflect your experience with comfort and fit? Why or why not? |
| **Daily Use & Routine Integration** |
| - 1. Does this item capture the challenges you’ve faced in using the splint daily? |
| - 1. Is there anything missing or unclear in how this item is worded? |
| **Social & Emotional Factors** |
| - 1. How do you feel about the way this checklist addresses social aspects of adherence? |
| - 1. Does this reflect your own experience, or would you phrase it differently? |
| **Financial & Accessibility Barriers** |
| - 1. When reading this item, does it make sense in the context of your personal situation? |
| - 1. Are there any financial or accessibility concerns not covered in this checklist? |
| **Healthcare Provider Guidance & Instructions** |
| - 1. Do you think the way the checklist describes the role of healthcare providers in adherence is accurate? |
| - 1. Did the instructions you received about using the splint match what is described here? |
| **Overall Checklist Evaluation & Refinement** |
| 1. **Now that you’ve gone through the checklist, do you think it fully captures your experience with using a splint (orthosis)?** |
| 1. Are there any statements that felt confusing, unclear, or not relevant to you? |
| 1. Are there any important factors affecting your adherence that you feel should be added? |
| **General Perception of Adherence** |
| 1. **How has the orthosis (splint) affected your daily life?** |
| 1. How do you personally define “adherence” when it comes to using your orthosis (splint)? |
| 1. What does “using it as prescribed” mean to you? |
| 1. Have you ever needed to modify how or when you use the splint (orthosis) to make it work better for you? Can you describe what changes you made and why? |
| **Closing Question** |
| 1. **Is there anything else about your experience with the orthosis that you think would be important for us to know and we did not cover it in the previous questions?** |
